# Book and Pamphlet Notices

**Published:** 1860-02

**Authors:** 


### BOOK AND PAMPHLET NOTICES.

The Obstetric Catechism: containing two thousand three hundred and forty-seven Questions and Answers on Obstetrics proper. By Joseph Warrington, M. D. One hundred and fifty Illustrations. Philadelphia: J. B. Lipincott & Co. 12mo. Pages, 445.

This little volume is intended as a *vade-mecuum* a reservoir of knowledge, on an important department of the science and art of medicine. The questions are in general well put, and the answers correct; and so long as the book is used as a *test* book, and not as a *text* book, it may be worthy a place in the library of the physician and student.

The Kansas City Medical and Surgical Review.

We have received the first number of this journal, a periodical of forty-eight pages, published bi-monthly, in Kansas City, Missouri, at $2.00 per annum, in advance. The mechanical execution of this work, commends itself at once to our notice, and on examination of its table of contents, and the matter of its pages fully sustains the favorable impression that its external appearance produces. It deserves to be amply sustained, and we hope such may be its experience. We hail with pleasure every indication of the "Westward march of Empire."

M. B. Maughs, M. D. and T. S. Case, M. D., are its Editors and Publishers.

---

To SUBSCRIBERS.—Correspondents and those sending us their names as subscribers for the *Journal*, will save us much trouble, and insure to themselves prompt attention to any requests contained in their letters, by giving their address in full. So many towns in different States have the same name, that when the name of the State is omitted, we are often entirely at a loss where to mail the *Journal*, or direct answers to letters.

Any subscribers who do not receive the *Journal* regularly, will confer a favor by giving us immediate notice of that fact and if the fault rests with us, it will at once be corrected.

---

☞ The Rush Medical College Clinic will be continued every Saturday, at 3 o'clock, during the Summer as usual.

All operations performed there gratuitously. The Alumni and friends of the Institution are invited to attend.

Spies and irregular practitioners will be excluded as hertofore

---

☞ A valuable communication from Springfield, Ill., on Spine Bifida, has been mislaid. We will feel obliged for another copy.

---

☞ The article in the January number on *Rhinoplasty* operations, the title of which was omitted, was by the senior editor.

---

OBITUARY.—Died at Pecatonica, Winnebago Co., Ill., of typhoid fever, Dr. A. W. Butler, aged 40 years.

